# Late immune consequences of combat trauma: a review of trauma-related immune dysfunction and potential therapies

**DOI:** 10.1186/s40779-019-0202-0

**Published:** 2019-04-24

**Authors:** Kelly B. Thompson, Luke T. Krispinsky, Ryan J. Stark

**Affiliations:** 1 Division of Critical Care Medicine, Department of Pediatrics, Vanderbilt University School of Medicine, 2200 Children’s Way, Nashville, TN 37232 USA; 2 Division of Pediatric Critical Care Medicine, Department of Pediatrics, Uniformed Services University, Naval Medical Center Portsmouth, Portsmouth, VA 23708 USA

**Keywords:** Trauma, Sepsis, Compensatory anti-inflammatory response syndrome, Persistent inflammation-immunosuppression and catabolism syndrome, Immune dysfunction

## Abstract

With improvements in personnel and vehicular body armor, robust casualty evacuation capabilities, and damage control resuscitation strategies, more combat casualties are surviving to reach higher levels of care throughout the casualty evacuation system. As such, medical centers are becoming more accustomed to managing the deleterious late consequences of combat trauma related to the dysregulation of the immune system. In this review, we aim to highlight these late consequences and identify areas for future research and therapeutic strategies. Trauma leads to the dysregulation of both the innate and adaptive immune responses, which places the injured at risk for several late consequences, including delayed wound healing, late onset sepsis and infection, multi-organ dysfunction syndrome, and acute respiratory distress syndrome, which are significant for their association with the increased morbidity and mortality of wounded personnel. The mechanisms by which these consequences develop are complex but include an imbalance of the immune system leading to robust inflammatory responses, triggered by the presence of damage-associated molecules and other immune-modifying agents following trauma. Treatment strategies to improve outcomes have been difficult to develop as the immunophenotype of injured personnel following trauma is variable, fluid and difficult to determine. As more information regarding the triggers that lead to immune dysfunction following trauma is elucidated, it may be possible to identify the immunophenotype of injured personnel and provide targeted treatments to reduce the late consequences of trauma, which are known to lead to significant morbidity and mortality.

## Background

In modern global conflicts, asymmetric warfare has led to a number of injuries in combat personnel that differ from prior conflicts. Since the start of Operation Iraqi Freedom (OIF) in 2003, the use of improvised explosive devices (IEDs) and ambushes with rocket propelled grenades has led to an increase in the number of personnel wounded or killed by explosions and fewer casualties resulting from gunshot wounds compared to prior conflicts [1]. During the Vietnam conflict, 16% of injuries were in the head or neck region, and 13.4% were in the thoracic area. OIF witnessed a significant shift in the injury pattern, with more than 30% of injuries occurring in the head or neck region and only 5.9% occurring in the thoracic region, a trend that continues in current conflicts due to improvements in personnel and vehicular body armor. Armor use has also led to a decrease in the number of deaths as a result of gunshot wounds, which were down to 4.8% [1, 2]. In data examining the timing and causes of death for patients surviving transport to a forward deployed combat surgical hospital in Iraq from 2007 to 2008, head injury and truncal and/or extremity hemorrhage were the cause of death in 77% of all patients. Most deaths occurred in the acute phase of care, with less than 10% occurring more than 7 days after admission. Of unexpectant deaths with higher preventability scores, hemorrhage was the leading cause of death (64%), followed by multi-organ dysfunction syndrome (MODS) (20%), hypoxia (13%), and brain injury (3%). These patients had lower mean injury severity scores (ISS) and were less likely to have severe head injuries; however, they had biochemical evidence of severe injuries as evidenced by significant acidosis, coagulopathy, and hypotension on presentation and the majority required massive transfusion (> 10 units of red blood cells in 24 h). The survival of combat-related injuries is in part due to the need for post-injury surgical care, where amputations accounted for 11% of all wounds, and 12% of all casualties suffered spinal cord injury [3]. Though there have been significant improvements in combat casualty care, with a focus on preventing deaths from hemorrhage, the relatively high incidence of surgical care and post-injury MODS contributes to the burden of morbidity and mortality in those who survive the initial trauma [4]. The associated morbidity and mortality is multifactorial, among which dysregulation of the immune system is an important contributing factor. Immune dysregulation leads to an increased risk for late onset sepsis and infection, acute respiratory distress syndrome, and delayed wound healing in addition to late MODS [5, 6].

## Medical stabilization after combat-related injury

An important factor in the development of MODS and immune dysregulation in the setting of trauma is the need for post-injury surgical intervention. Given the increased incidence of explosive injuries in modern combat that lead to multiple injury patterns, it is useful to utilize explosive injuries as a model for understanding the complex nature of trauma-induced MODS. Primary blast injuries caused by the overpressure wave passing through the body lead to damage at the air-fluid interfaces of the tympanic membrane, the lungs, and intestine, and may lead to hollow viscus rupture and internal hemorrhage that can be difficult to identify upon initial triage [7]. Secondary blast injuries occur as a consequence of high-energy projectiles being released from the explosive, leading to extensive tissue injury or loss and wound contamination. Tertiary blast injuries result from blunt trauma sustained when the casualty is blown back from the explosion into other objects. Quaternary blast injuries involve injuries sustained due to the chemical nature of the blast, including burns and inhalation injuries, as well as when other objects are blown onto the casualty, leading to crush injuries and perhaps further penetrating injuries. Injuries from explosives therefore present with numerous complex wounds that are likely to be contaminated and are associated with extensive tissue damage or loss and compounded by microvascular trauma as well. Based on the NATO triage doctrine, following initial stabilization and basic hemorrhage control by combat medics (Role 1 care), the injured are moved to Role 2 facilities at forward operating bases (FOBs) for the provision of damage control resuscitation (DCR), which includes further hemorrhage control, decontamination or debridement of wounds, and limb or life-preserving surgeries, including exploratory laparotomy with temporary wound closure and the application of negative pressure wound therapy [8]. Administration of blood products and fluids is continued in a balanced manner to restore circulating volume and organ perfusion while allowing for “permissive hypotension”. In recent conflicts, approximately 8 to 10% of military casualties undergo massive transfusion, receiving more than 10 units of blood in the first 24 h after injury. Such large volume transfusion has been associated with immune suppression, coagulopathy, acidosis, organ dysfunction and hypothermia [9, 10]. These effects are further compounded by uncontrolled pain associated with the injuries of the wounded, which has been shown to induce an inflammatory state leading to hypercoagulation, an increased metabolic demand of tissues, and impaired immune function [9, 11]. Patients are then moved to a Role 3 forward-deployed Level 1 trauma-equivalent hospital where DCR is continued. The injured undergo further operations of their wounds and are managed in a critical care facility with the aim of restoring physiological function and limiting the effects of prolonged operative times, massive transfusion, and multiple complex injuries. It is here that the late effects of trauma may begin to develop. Once stabilized enough for transport, typically within 7 days after injury, patients are evacuated to Role 4 facilities for more definitive or specialized care and rehabilitation [9, 12, 13]. Within the first few weeks after injury, patients may undergo multiple surgeries, including initial surgeries for decontamination and hemorrhage control, followed by further debridement, grafting, amputations, primary closures and reconstructions. Despite timely and appropriate medical and surgical care, the consequences of immune dysfunction after the initial trauma may persist and lead to further complications for survivors.

## Mechanisms of post-injury immune dysfunction

**Fig. 1:**
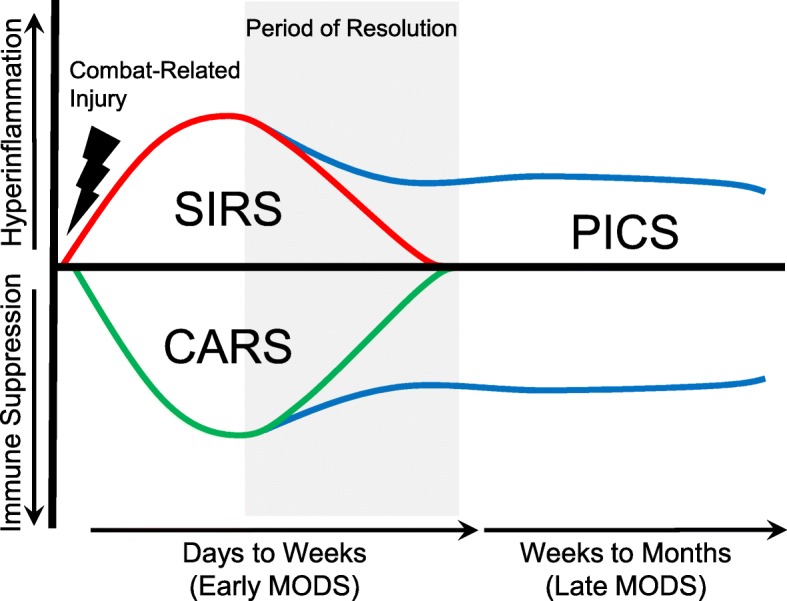
Temporal association of immune dysfunction syndromes. After an initial combat-related injury, there is the development of a hyper-inflammatory response, termed the systemic inflammatory response syndrome (SIRS), and an immune suppressing response, termed the compensatory anti-inflammatory response syndrome (CARS). These two responses happen within minutes to days, occurring nearly simultaneously, and it is during these initial inflammatory phases that death from early multi-organ dysfunction syndrome (MODS) may occur. As both the pro-inflammatory and anti-inflammatory responses resolve, there is a period of resolution, typically within days to weeks, that allows for the return to homeostasis and survival after the injury. However, in a percentage of injured patients, the pro-inflammatory and/or anti-inflammatory responses never resolve, leading to a period of chronic critical illness termed persistent inflammatory-immunosuppressive and catabolic syndrome (PICS). This occurs in patients who have been critically ill for longer than 14 days with significant lymphopenia and chronic inflammation. PICS may persist for months and lead to the risk of developing later MODS and secondary infections with subsequent morbidity and late mortality

The initial concepts of immune dysregulation and dysfunction came from a consensus meeting in 1991 describing the whole-body response to an infectious or injurious stimulus, which came to be known as the systemic inflammatory response syndrome (SIRS) [14]. These concepts later evolved to incorporate the response of counter-regulatory mechanisms designed to dampen the initial pro-inflammatory signal, termed the compensatory anti-inflammatory response syndrome (CARS) [15]. The temporal association of SIRS and CARS was initially conceptualized to happen in the sequence of SIRS and then CARS, but this belief has been challenged by a model demonstrating more overlap between the two responses [16]. In addition, our more recent understanding of the complex integrated pro- and anti-inflammatory responses to injury has also led to the acknowledgement of a protracted form of immune dysregulation, termed persistent inflammation-immunosuppression and catabolism syndrome (PICS) (Fig. 1) [17].

While the clinical and temporal evolutions of SIRS, CARS and now PICS have undergone revisions as our understanding of their associated immune phenotypes has evolved, the underlying concepts of pro- and anti-inflammatory responses have remained similar since they were first postulated. Following the initial trauma, a host of immune mediators are released by various cells and tissues within the body to activate the immune system and promote a pro-inflammatory state through the expansion and recruitment of various cell lines with the goal of preventing or combating infection and eliminating dead or dying tissue. This pro-inflammatory state is carefully balanced with a compensatory anti-inflammatory response to limit further tissue damage, preserve organ function, and ultimately quiet the pro-inflammatory state and return the body to homeostasis. In severe trauma, there may be an exaggerated pro-inflammatory state, which leads to further injury and rapid multiple organ failure. This may be combined with or followed by an exaggerated and prolonged compensatory anti-inflammatory response, which is associated with immunosuppression through lymphocyte dysfunction and apoptosis, down-regulation of monocyte human leukocyte antigen (HLA) receptors, monocyte deactivation, and unbalanced production of cytokines and anti-inflammatory mediators. These effects place injured patients at risk for late complications, secondary to susceptibility to infection and inability to clear infections [18].

### Damage-associated molecular patterns and cytokines

Recent studies have suggested that damage-associated molecular patterns (DAMPs) are key to the initiation and continuation of both SIRS and CARS and may play a critical role in both the “one-hit” and “two-hit” models for the development of MODS as well as the subsequent development of PICS [19]. Under these conditions, endogenous molecules, such as cytokines (tumor necrosis factor, interleukin-1 beta) or alarmins (interleukin-1 alpha, high mobility group box 1, S100), are released from activated or injured cells to promote a host response, and their presence has been linked to outcomes after trauma [20, 21]. More specifically, cytokines are released when pattern recognition receptors, the typical receptors to which DAMPs bind, are activated on immune cells, while alarmins, constitutively active molecules produced by somatic cells, are released when cells undergo necrosis or apoptosis [22]. The release of alarmins, such as high mobility group box 1 (HMGB1), has been demonstrated to occur as soon as 30 min after injury. This rapid release in response to trauma is in contrast to the delayed release demonstrated in the setting of severe infections [23–25]. While the production and release of these molecules is intended to recruit cells to the site of injury and contain its effects, they also alter the response to later infectious or injurious challenges, termed immunotolerance [26]. This tolerant phenotype was first described in trauma patients in the mid-1990s, where monocytes isolated from injured patients had a reduced cytokine response to ex vivo stimulation of endotoxins [27]. Though significant debate still exists regarding the mechanisms and effects of immunotolerance following injury or infection, population-based studies have demonstrated a correlation between the presence of endotoxin tolerance and the development of organ dysfunction [28, 29]. One of the more important cytokines associated with an immunotolerant phenotype is interleukin-10 (IL-10). This was first shown in IL-10 knockout mice that demonstrated an impaired tolerant phenotype to repeated endotoxin challenge [30]. Persistently elevated IL-10 levels in the plasma have also been correlated with a worse outcome in patients with sepsis and have been associated with the development of secondary complications after burn injury and trauma [31–33]. More specific to combat-related injuries, higher IL-10 levels have been shown in those who develop MODS, as well as in non-survivors compared to survivors [34]. Similar to IL-10, elevated levels of transforming growth factor β (TGF-β), another anti-inflammatory cytokine, have been shown to correlate with the severity of injury and the development of secondary infections [35]. Comparatively, for those who survive the initial injury, an excessive predominance of pro-inflammatory markers compared to anti-inflammatory markers has been associated with poor wound healing, suggesting a temporal imbalance in immune function recovery and specific trauma-related outcomes [36].

### Innate and adaptive immune dysfunction

Immune dysfunction after injury has been shown to impact both the innate immune system, which is able to immediately respond without reprogramming or differentiation, and the adaptive immune system, which requires secondary activation and programing via cell-cell contact [37]. One classical feature of immune dysfunction after systemic inflammation is a reduced expression of human leukocyte antigen DR (HLA-DR) on peripheral blood mononuclear cells, which are innate immune cells. This reduced HLA-DR expression is associated with impaired antigen presentation [38]. As early as the 1980s, it was recognized that major trauma results in decreased expression of HLA-DR on monocytes and was linked to an increased risk for infection during the recovery period, leading to late morbidity and mortality [39]. These findings have been confirmed in multiple subsequent studies, which have suggested that both a more robust initial inflammatory reaction along with the inability to recover HLA-DR expression predispose and prognosticate trauma patients to subsequent development of sepsis [40, 41]. In addition, reduced HLA-DR expression has been observed within 24 h after surgery and can be restored through the application of granulocyte-macrophage colony stimulating factor (GM-CSF) and interferon-gamma (IFN-γ) [42]. Continued suppression of monocyte HLA-DR expression has also been correlated with a worse outcome in patients with sepsis [43]. Though monocytes and the tissue variant of monocytes, known as macrophages, have been the stereotypical innate immune cells to demonstrate immune dysfunction after trauma, other innate immune cells have been shown to have impaired activity, including neutrophils, dendritic cells and natural killer cells [19]. The immunosuppressive phenotype displayed by these innate immune cells typically involves decreased phagocytosis, decreased cytokine production, decreased cytotoxic function and an overall susceptibility for apoptosis [44].

**Fig. 2:**
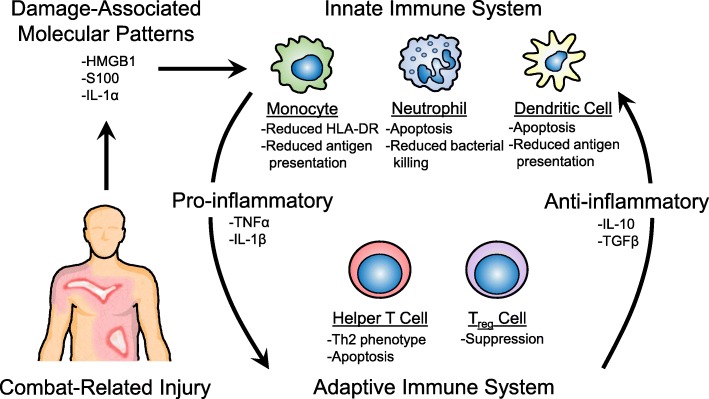
Interactions of the innate and adaptive immune systems in response to trauma. Immediately following injury, damaged tissues release damage-associated molecular patterns (DAMPs) and in response, residing innate immune cells release pro-inflammatory cytokines. These signals help recruit other innate immune cells to the site of injury in an attempt to contain the deleterious effects of the injury. However, in severe injuries, the immune response goes beyond the local site of injury and leads to systemic inflammation. To reduce the impact of systemic inflammation, the adaptive immune system, primarily through the suppression of regulatory T cells (T_reg_), releases anti-inflammatory cytokines and other signals that impede the immune system as it tries to continue the pro-inflammatory response. This manifests as apoptosis of innate immune cells and decreased antigen presentation (HLA-DR on monocytes), as well as apoptosis and the anergy of helper T cells causing leukopenia. In the maladaptive state, preponderance of this anti-inflammatory, immune suppressing phenotype leads to the consequences of CARS and PICS. The general effect of a chronic inflammatory state on immune systems in response to injury is listed below their respective cell types. For a general review of the immune system and inflammation, the reader is referred to a review by Spiering [37]

The inactivation of monocytes after surgery, trauma, and infections further propagates immune dysfunction through alterations in T lymphocyte function. Lymphopenia itself is known to occur after severe injury, and a lack of lymphocyte recovery is known to impact survival [45]. Beyond changes in lymphocyte number, circulating effector T lymphocytes also change from a pro-inflammatory Th1 phenotype to an anti-inflammatory Th2 phenotype [46]. This change in phenotype is partly due to suppression by regulatory T cells, which are important mediators of IL-10 and TGF-β production. The impairment of effector helper T lymphocytes after trauma also results in a reduction of interferon gamma (IFN-γ) production by Th1 polarized cells [47]. IFNγ serves a key function in stimulating increased antigen presentation and anti-pathogen activities of innate immunity cells [48]. After major surgery, while the number of effector T cells decreases, the number of regulatory T cells remains relatively unchanged [49]. These regulatory T cells express the receptor programmed death 1 (PD-1), which can act as a negative regulator on other immune cells, particularly antigen-presenting cells expressing the programmed death ligand 1 (PD-L1) [50]. A high expression of PD-1 on T lymphocytes has been correlated with the severity of illness after major trauma [51]. Beyond T lymphocytes, B lymphocytes are also affected, resulting in impaired antibody production as well as apoptosis [44]. A summary of the initial combat injury and the subsequent major pro- and anti-inflammatory responses is shown in Fig. 2.

While the proposed mechanisms of immune dysfunction mentioned here are not exhaustive and likely involve a complex and dynamic host of responses to curtail the integrated inflammatory response, it is increasingly clear that trauma and the necessary associated surgeries alter the immune system. In those injured personnel who develop more aberrant phenotypes of immune dysfunction, there is a higher risk of developing late complications of the initial injury.

## Late complications of altered immune function after injury

Despite the early and aggressive medical management of patients as they are moved through various levels of care, alterations in immune function following trauma can place patients at risk for late complications of trauma. In addition, the inability to have sufficient resolution from SIRS or CARS can lead to the development of PICS. The consequences of these altered immune phenotypes can lead to impaired wound healing, late onset sepsis, MODS, and acute respiratory distress syndrome (ARDS) [5, 6].

### Wound infections and delayed wound healing

In a study from 2010 investigating the incidence of wound infections from wounded personnel arriving at a Role 4 facility 1 week after injury from combat operations in Afghanistan and Iraq, approximately 40% of the wounds biopsied were infected or critically contaminated as defined by wound tissue biopsy cultures with greater than 10 × 10^5^ CFU/g of biopsied tissue at admission. Of infected wounds, gram-negative bacteria predominated, with *Acinetobacter baumannii* being the most common pathogen throughout the study period. This finding was consistent with other reports of predominance in orthopedic wounds and osteomyelitis [12]. In combat wounds treated at a referral facility within 1 week of injury, nine wounds (24%) in five patients (20%) demonstrated impaired healing, including five delayed wound closures in three patients and four wound dehiscences in two patients, despite appropriate surgical debridement. Delays in wound closure were made due to concerns about infection (*n* = 3) or severe systemic illness (*n* = 2). Delayed wound healing was found to be associated with increased serum concentrations of multiple inflammatory mediators, including IL-6, IL-8, and matrix metalloproteinase-7 (MMP-7). Increased effluent concentrations of IL-6, IL-8, and macrophage inflammatory protein 1 alpha (MIP1α) were also predictive of critical contamination of wounds prior to closure. Each of these bio-markers was also independently associated with wound outcome. Many of these patients were critically ill on admission with a mean (±SD) ISS of 21 ± 12 and a mean Acute Physiology and Chronic Health Evaluation (APACHE) II score of 7 ± 5 on admission. The critical interplay between systemic and local inflammation and wound bacterial burden likely contributes to wound outcome. The balance of chemokines, cytokines, and matrix metalloproteinases that are necessary for appropriate wound healing may be altered by the presence of bacteria at the wound site. Furthermore, this balance may be altered by a dysregulated immune response secondary to injury, with a higher risk of immunosuppression-associated infection or failure to clear the bacterial burden and chronic local inflammation at the tissue bed [6].

Although there is a high incidence of blast injuries sustained by combat personnel, only a small percentage (3 to 5%) suffer burn injuries [52]. Despite this, infected burns are a difficult subset of wounds to manage as wound infection leading to sepsis is the most common cause of mortality in burn patients after burn injury and an important component to delayed wound healing. Furthermore, due to the disruption of the protective epithelial layer of skin, burn patients are at risk for invasive bacterial and fungal infections. Clinicians must have a high index of suspicion for infection as thermal injury-induced hyperpyrexia, immune suppression, and systemic inflammatory response syndrome may alter the typical presenting features of infection and make infection difficult to control [53]. *Pseudomonas aeruginosa*, *Klebsiella pneumoniae*, *Escherichia coli* and *Staphylococcus aureus* are independent predictors of mortality, with *S. aureus* being a major cause of septicemia in burn patients [54]. Furthermore, the gram-negative *P. aeruginosa*, *E. coli*, and *K. pneumoniae* are also associated with failure to heal infected burn wounds [53]. Additionally, skin grafting is a common surgical procedure for the management of burns; however, given the presence of co-existing injuries, amputations, and the critical illness of patients, suitable donor sites are difficult to obtain and harvest, potentially leading to delayed healing and an increased risk of infection [9].

### Late onset sepsis and multi-organ dysfunction syndrome

Sepsis is defined as life-threatening organ dysfunction caused by a dysregulated host response to infection [55]. Sepsis is a major cause of morbidity and mortality after trauma, as alterations in immune function following trauma contribute to an increased susceptibility and impaired ability to combat infection through the modification of both the innate and adaptive immune functions. Furthermore, the development of MODS is often associated with infection and is the most common cause of late death in trauma patients who survive past the first 24 to 48 h of resuscitation [34]. Following major trauma, SIRS is initiated by the activation of the innate immune response. This is often soon followed by CARS, which is controlled by the adaptive immune system and was previously thought to occur approximately 5 to 15 days after trauma [56]. However, more recent research has demonstrated that SIRS and CARS may occur at the same time with the robustness of each response dependent on a variable milieu of cytokines and other mediators [17, 57]. Massive trauma can lead to an accelerated and substantial inflammatory response and severe SIRS, independent of infection, leading to a “one-hit” initiation of MODS [19, 58]. Patients with less severe trauma may develop late MODS due to new surgical stress, general anesthesia, transfusion of blood products, infection, or ischemia/reperfusion injury triggering the reactivation of the inflammatory response in a “two-hit” model of MODS [19, 58]. A key inciting event in the development of MODS and sepsis may be the transfusion of blood products. Studies have demonstrated that the transfusion of red blood cells to an already primed immune system leads to a significant increase in IL-10 and TNF-α production by monocytes, which can have deleterious effects following injury or infection [59]. These effects are likely a result of DAMPs, residual white blood cells, and other soluble and insoluble mediators within the donor blood that contribute to a cascade of transfusion-related immunomodulation (TRIM), although the exact mechanisms remain difficult to elucidate. Regardless, the transfusion of red blood cells has been associated with worsening organ dysfunction, increased rates of infection, and increased mortality [10, 19, 60].

CARS typically occurs in conjunction with late onset MODS, as immunosuppression increases the potential for hospital acquired infections via immune-inflammatory dysregulation in which the balance of pro-inflammatory and anti-inflammatory mediators is disrupted [56]. Furthermore, it has been postulated that cytokines produced during this period of immune dysregulation may actually favor or promote the growth of bacteria [34]. According to the trauma register of the German Society of Traumatology, more than 6% of civilian trauma patients with multiple injuries develop septic complications, with 20% of the patients developing multiple organ failure [61]. Among combat personnel admitted to a Role 3 facility during Operation Iraqi Freedom, 56 of 211 (26.5%) developed infections, with 84% of cases having wound infections followed by 38% with bacteremia and 21% with pneumonia. Infection was more likely with those patients having had surgery prior to admission, higher ISS, and injuries qualified as blast, abdominal, soft tissue, ≥ 3 injury locations, or loss of limb. *S. aureus*, *E. coli*, *P. aeruginosa* and *A. baumannii* were the dominant causative organisms of infection, with many demonstrating multidrug resistance [62]. Sepsis and other nosocomial infections increase the risk of late onset MODS, which carries a significant mortality burden. In another study of combat-related trauma patients with and without sepsis, of 56 casualties with severe trauma who developed sepsis, 47 developed MODS and 32 died. Of the 20 matched casualties with severe trauma and no evidence of sepsis, 8 developed MODS and 4 died, demonstrating a 2.5-fold higher mortality when trauma is complicated by sepsis [34]. In combat-related burn patients, the presence of *K. pneumoniae* bacteremia was independently associated with an increased risk of mortality and increased ventilator days [63]. According to the American Burn Association National Burn Repository, the primary cause of death in burn patients with sepsis is multi-organ failure (27.5%), followed by pulmonary failure/sepsis (11.3%) and burn wound sepsis (4%), with a higher total body surface area involvement associated with an increased risk of sepsis development and mortality [64].

### Acute respiratory distress syndrome

Acute respiratory distress syndrome (ARDS) is the most frequent manifestation of MODS following trauma, with 12 to 25% of injured patients ultimately developing the syndrome. Trauma patients who develop ARDS along with MODS have mortality rates as high as 50 to 80%; however, the attributable mortality to ARDS alone in this population has been difficult to delineate given the severity of co-existing injuries. Additionally, ARDS causes significant morbidity in the trauma population, demonstrating increased rates of complications, longer hospital and ICU lengths of stay, and increased hospital costs [65]. ARDS has been shown to have varying patterns of onset within trauma cohorts with distinct risk factors for each pattern. In a 2013 study utilizing latent class analysis examining the timing of onset of ARDS in patients with trauma, 2 major phenotypes were identified: early onset ARDS (occurring < 48 h after trauma) and late onset ARDS (occurring > 48 h after trauma). Early onset ARDS was associated with an increased severity of thoracic trauma score, more severe early hypotension, and increased red blood cell transfusion during the initial resuscitation, suggesting that early onset ARDS may be characterized by higher ISS and severe hemorrhagic shock necessitating the transfusion of blood products, which is consistent with a “one hit” model of MODS and immune dysfunction. Late onset ARDS was hypothesized to be associated with progressive MODS and nosocomial infections consistent with the “two-hit” model of MODS, in which dysfunction of the innate and adaptive immune systems plays a role in inappropriate immunosuppression, leading to an increased risk of nosocomial infections. Despite the two phenotypes, there was no significant difference in mortality between early- and late-onset ARDS [66]. In one study from 2016, of 4679 mechanically ventilated US combat casualties from Operation Iraqi Freedom/Enduring Freedom, ARDS was identified in 3.3% and was associated with higher military-specific ISS as well as hypotension and tachycardia at initial presentation. ARDS was also an independent risk factor for death (OR 1.99) [67]. Additionally, large volumes of plasma and crystalloid infusion have been identified as independent risk factors for the development of ARDS in combat personnel [68]. In a study examining the incidence and mortality of ARDS in combat-related burn patients, 32.6% of mechanically ventilated burn patients developed ARDS with an overall mortality of 16.5%. However, mortality increased in accordance with ARDS severity, with severe ARDS demonstrating 43.8% mortality and a 9-fold increased odds of death. Predictors for the development of moderate or severe ARDS were inhalation injury, higher ISS, pneumonia, and the transfusion of fresh frozen plasma (FFP). [69]. A recent study has demonstrated that the presence of mitochondrial DNA (mtDNA) DAMPs from blood products is associated with the development of ARDS with FFP and platelets having the highest amounts of mtDNA fragments prior to transfusion. Following transfusion, patient serum concentrations of mtDNA fragments increased linearly, with the serum quantity at 24 h after transfusion being a predictor for the occurrence of ARDS (9.9 vs 3.3) [70].

### Persistent inflammation-immunosuppression and catabolic syndrome

Recently, with the advances provided by critical care medicine, more patients are surviving beyond the well-established SIRS, CARS, and early MODS phenotypes and developing a chronic critical illness. This chronic critical illness is characterized by ongoing protein catabolism and a combination of inflammation and immunosuppression termed persistent inflammation-immunosuppression and catabolic syndrome (PICS), which serves as a prolonged form of MODS with late-term mortality [57]. PICS was characterized by Gentile and Moore et al. [17] in 2012 as ICU stay > 14 days, c-reactive protein ≥150 μg/dL, total lymphocyte count < 0.8 × 10^3^/μL of blood, weight loss > 10% during hospitalization or body mass index < 18, creatinine height index < 80%, albumin level < 3.0 g/dL, prealbumin level < 10 mg/dL, and retinol binding protein level < 10 μg/dL. PICS patients suffer from increased long-term mortality and have increased morbidity associated with manageable organ dysfunctions, poor wound healing, recurrent nosocomial infections, delirium, psychosocial stress, and prolonged rehabilitation needs with a decreased likelihood of returning to pre-insult functional status. Recent research has demonstrated that SIRS and CARS may occur and proceed concurrently for prolonged periods of time leading to PICS and that in addition to the mechanisms previously discussed, myeloid-derived suppressor cells (MDSCs) may also play a critical role in the development of PICS by augmenting both the immunosuppressed and pro-inflammatory state [17]. Following severe trauma or infection, granulocytes rapidly demarginate from the bone marrow and lymphocytes undergo massive apoptosis, creating space for hematopoietic progenitor production in an ‘emergency myelopoiesis-granulopoiesis’ [17]. Production in these disease states is shifted towards myelopoietic precursors, including MDSCs, with the degree of expansion and persistence of MDSCs being proportional to the severity of the inflammatory insult. MDSCs are both pro-inflammatory and immunosuppressive through their interaction with T-cells and the production of various cytokines. Though the precise incidence and evolution of PICS after combat injury has not been studied, injured combat personnel may suffer from a milder form of PICS as identified by chronic manageable organ dysfunction [71]. Stewart et al. [71] demonstrated that of the combat injured personnel admitted to an ICU, the ISS at admission was consistently associated with an increased risk of development of hypertension, coronary artery disease, diabetes mellitus, and chronic kidney disease and at a higher rate than would be expected when compared to military controls. The development of these chronic diseases is likely, at least in part, driven by a chronic inflammatory response initiated by the initial injury and subsequent medical care, as a number of pro-inflammatory cytokines have been implicated in the development of hypertension, diabetes mellitus, coronary artery disease, and chronic kidney disease [71].

## Immunomodulatory therapies after combat injuries

Despite the overwhelming presence of both systemic pro-inflammatory and compensatory anti-inflammatory responses after injury, treatment to curb the exaggerated phenotypes remains elusive. The reasons for the absence of targeted therapy are numerous; however, the crux of the issue lies in appropriately identifying the dynamic immunophenotype of a patient after injury. While the pro-inflammatory state happens immediately after injury, work from the late 1990s showed that tolerance to endotoxin challenge could happen as soon as 90 min after traumatic injury [72]. While this may be an appropriate response to dampen the initial pro-inflammatory cascade, persistence of an anti-inflammatory phenotype after day 3 of illness has been associated with higher mortality [43]. Thus, it seems reasonable to prevent or attempt to reverse the anti-inflammatory phenotype before the development of immune dysfunction. Several therapies have been utilized, though the results have been mixed.

### Granulocyte-macrophage colony stimulating factor and granulocyte colony stimulating factor

GM-CSF and granulocyte colony stimulating factor (G-CSF) have been suggested as therapies to reverse the effects of immunosuppression. In a randomized, double-blinded trial of 60 patients who had suffered traumatic brain injury or cerebral hemorrhage, the early application of G-CSF (300 μg/day) was associated with a reduced incidence of bacteremia, though not on other nosocomial infections or mortality [73]. In another randomized control trial of 38 patients with sepsis-induced immunosuppression, defined as reduced monocyte human leukocyte antigen-DR (mHLA-DR) expression, patients received either a placebo or GM-CSF (4 μg/kg/day) [74]. Those in the GM-CSF group had a reduced duration of mechanical ventilation and an improved ex vivo monocyte cytokine response to bacterial endotoxin. Though data on the use of G-CSF during conflict is limited, it has been utilized to treat the myelosuppressive effects of mustard gas during the Persian Gulf War, suggesting that it could be offered in forward operating areas to aid in recovery [75]. However, these results are tempered by a meta-analysis of both G-CSF and GM-CSF, demonstrating that while there was a quicker reversal of sepsis in patients who received therapy, there was no improvement in 28-day survival [76].

### Interferon-gamma

IFN-γ is a cytokine important for the regulation of T cell function. Early animal studies, such as one looking at infection mortality after hemorrhagic shock, showed that IFN-γ prophylaxis could reverse the immunosuppressive phenotype after injury [77]. A later randomized, multicenter trial tested this hypothesis in severely injured patients through the preventive application of daily subcutaneous injections of IFN-γ (100 μg) for 21 days. While early mortality was not affected, mortality from infection was reduced in the IFN-γ treatment group after 7 days [78]. However, a later study in burn-injured patients receiving IFN-γ prophylaxis for 10 days showed no difference in infection rates compared to placebo controls [79]. Though the application of IFN-γ after combat-related injuries has not been tested, issues could possibly arise from late complications related to the treatment, with a specific focus on wound healing, as animal studies have suggested that systemic IFN-γ treatment can impair wound healing [80]. Conversely, data showing that in dehisced wounds from combat-related injuries, IFN-γ expression is suppressed compared to wounds that heal appropriately, suggesting that either high or low levels of IFN-γ can alter the inflammatory response related to proper wound healing [36].

### Intravenous immunoglobulin

The use of pooled intravenous immunoglobulin (IVIG) has been proposed as an immunomodulator for some time. The concept behind its use is multifactorial, including receptor blockade, antigen binding, and opsonization. Over the past several decades, numerous studies have been conducted examining the utility of either polyclonal or antigen-specific monoclonal IVIG in the treatment of sepsis. In aggregate, systemic reviews and meta-analyses have led to no definitive conclusion about the efficacy of IVIG in septic patients [81]. However, within the more specific post-surgical population, the use of IVIG has improved sepsis-mediated ICU outcomes, especially when combined with appropriate antibiotic therapy [82, 83]. In addition, one study examined the prophylactic application of IVIG therapy in trauma patients. This randomized study tested the use of polyclonal IVIG compared to albumin given in escalating doses (250 to 1000 mg/kg/day) on hospital days 0, 2, 3 and 6 after admission for trauma. These patients also received penicillin prophylaxis on hospital days 0 through 4. While there were no deaths related to infections in either group, the group that received IVIG had a lower rate of nosocomial pneumonia and non-catheter infections [84]. Though the application of IVIG after combat-related injuries to prevent immunologically induced organ dysfunction has not been tested, IVIG has been used in deployed settings as a treatment for autoimmune diseases, suggesting the feasibility of such prophylactic use in combat areas [85]. The feasibility of IVIG use in deployed settings is further enhanced through the development of lyophilized IVIG, which has a similar efficacy, yet a longer shelf life, that could be maintained within forward operating areas [86].

### Interleukin-10 and transforming growth factor β

Despite the association of IL-10 and TGF-β with an immunosuppressive phenotype, the application of IL-10 antagonism to correct immunosuppression after trauma or injury has not been fully tested. Animal models have suggested that the use of IL-10 or TGF-β blocking antibodies can improve survival in polymicrobial sepsis [87]. In addition, the combination receptor antagonism of IL-10 and TGF-β has led to improved control of parasitic vectors similar to those observed in veterans who served in Middle Eastern conflicts, suggesting an additional potential benefit of IL-10 and TGF-β antagonism to improve immune function [88, 89]. Currently, data supporting the clinical use of anti-IL-10 antibodies are limited. Its use has only been tested in a single pilot study looking at IL-10 antagonism in patients with systemic lupus [90]. This is in contrast to TGF-β blockade, which has received significant interest within cancer immunology, with several small molecule inhibitors and antibodies in development [91]. The successful application of such therapeutics to combat-injured personnel to reverse immunosuppression remains unknown.

### Interleukin-7

While the application of the previously mentioned therapies has the largest amount of clinical evidence surrounding their use as immunomodulators in the post-injured or infected, other therapies are currently under investigation. One such therapy is interleukin-7 (IL-7). This endogenous anti-apoptotic cytokine has a main function in supporting the proliferation and survival of effector T cells [92]. Preclinical studies have supported the use of recombinant IL-7 as an immunostimulant to improve survival in animal models of sepsis [93, 94]. This has led to a recent trial of human recombinant IL-7 therapy to patients who had evidence of lymphopenia and persistent vasoactive medication requirements in the setting of sepsis [95]. Though the trial was underpowered to detect clinical differences, recovery in T cell counts and function was noted in the IL-7 group, and this effect persisted for several weeks after the completion of therapy, suggesting that a limited early application may have longer-lasting effects.

### Thymosin α1

Thymosin α1 is a peptide derived from thymic epithelial cells that has both immunostimulating and immunotolerizing effects on antigen presenting cells and T cells. Its use in humans as an immunomodulator dates back to the 1970s when it was used as a therapy to treat immunodeficiency in athymic patients [96]. The immunomodulatory effects eventually led to its development as a commercially available therapy, called Thymalfasin, which was tested as an adjuvant therapy in hepatitis and cancer [97, 98]. Its properties further led to the investigation of thymosin α1 as an adjuvant in sepsis. A recent systematic review of 19 clinical trials demonstrated that thymosin α1 offered daily during sepsis showed benefits with regard to improved T cell counts, reduced cytokinemia, and a reduction in mortality risk ratio to 0.59 [99]. There have been no studies examining the effectiveness of thymosin α1 in forward operating areas but given that it is supplied as a lyophilized powder that can be injected subcutaneously, its application in such areas would be testable.

### Programmed Death-1 and programmed death Ligand-1

Ameliorating T cell and macrophage dysfunction after injury has also been examined by targeting the programmed death-1 (PD-1) and programmed death ligand-1 (PD-L1) axis. Using animal models of sepsis, the application of PD-1 or PD-L1 antibodies around sepsis initiation was associated with reduced leukopenia and improved survival [100–102]. In humans, treatment of blood with anti-PD-1 or anti-PD-L1 antibodies from patients with sepsis or surgically mediated T cell suppression demonstrated decreased T cell apoptosis and increased IFN-γ production [103, 104]. Clinical trials of antibodies targeting PD-1 have been further employed in a variety of cancers as well as human immunodeficiency virus infection [105, 106]. Extrapolation of these efforts into treating patients with immunosuppression after sepsis led to a phase 1 clinical trial using an anti-PD-1 antibody (#NCT02576457); however, the trial was terminated in 2017. Though the pre-clinical data for modulating the PD-1/PD-L1 axis is promising, further data are needed to determine its potential role in reversing the immunosuppressed phenotype after combat-related injuries.

## Conclusion and future directions

The asymmetric warfare of modern conflicts has led to an increased number of wounded combat personnel injured by blast injuries due to the increased utilization of improvised and rocket propelled explosive devices. Patients who survive the initial trauma of injury and resuscitation are at risk for several late consequences of their injuries. Among these consequences, delayed wound healing, late onset sepsis and infection, multi-organ dysfunction syndrome, acute respiratory distress syndrome, and persistent inflammation-immunosuppression and catabolic syndrome are significant in their association with the increased morbidity and mortality of wounded personnel. These late consequences of trauma have been shown to be associated with a dysregulated immune system that leads to an immunosuppressed state with variable immunophenotypes. Promising research into determining the immune profiles of trauma patients to help personalize and target therapies may provide a potential avenue in preventing late complications and directing treatment [34, 107, 108]. Recent epigenetic work by Scicluna et al. [109] has demonstrated the ability to identify the immunophenotypes of sepsis patients according to the four molecular endotypes— Mars1, Mars2, Mars3, and Mars4. The Mars1 endotype was associated with increased 28-day mortality and was characterized by decreased expression of promoter genes for both the innate and adaptive immune systems, indicative of an immunosuppressed phenotype. The Mars2 and Mars4 endotypes were associated with genes involved in pro-inflammatory and innate signaling, while the Mars3 endotype was characterized by genes involved in the adaptive immune or T-cell pathways and was associated with the lowest risk of mortality. Trauma, like sepsis, induces changes in gene expression in accordance with epigenetic gene regulation principles. These modifications in DNA processing play a role in determining the immunophenotype of the injured [110]. Based on these results, PCR-based phenotype identification may soon be available for targeted treatment strategies [109]. Future research into immunomodulatory therapies and further advancements in resuscitation, including the use of artificial intelligence and machine learning to guide resuscitation efforts, will be necessary to improve the morbidity and mortality associated with the late consequences of trauma after combat-related injuries [111].

## Abbreviations

APACHE: Acute physiology and chronic health evaluation
ARDS: Acute respiratory distress syndrome
CARS: Compensatory anti-inflammatory response syndrome
CFU: Colony forming units
DAMP: Damage associated molecular pattern
DCR: Damage control resuscitation
FOB: Forward operating base
G-CSF: Granulocyte colony stimulating factor
GM-CSF: Granulocyte-macrophage colony-stimulating factor
HLA: Human leukocyte antigen
HMGB1: High mobility group box 1
IED: Improvised explosive device
IFN: Interferon
IL: Interleukin
ISS: Injury Severity Score
IVIG: Intravenous immunoglobulin
MDSC: Myeloid derived suppressor cells
mHLA-DR: Monocyte human leukocyte antigen-DR
MMP: Matrix metalloproteinase
MODS: Multi-organ dysfunction syndrome
mtDNA: Mitochondrial DNA
OIF: Operation iraqi freedom
PD: Programmed death
PICS: Persistent inflammation-immunosuppression and catabolic syndrome
SIRS: Systemic inflammatory response syndrome
TGF: Transforming growth factor
TNF: Tumor necrosis factor
TRIM: Transfusion-related immunomodulation
